# A machine learning approach for early prediction of gestational diabetes mellitus using elemental contents in fingernails

**DOI:** 10.1038/s41598-023-31270-y

**Published:** 2023-03-14

**Authors:** Yun-Nam Chan, Pengpeng Wang, Ka-Him Chun, Judy Tsz-Shan Lum, Hang Wang, Yunhui Zhang, Kelvin Sze-Yin Leung

**Affiliations:** 1 Department of Chemistry, Hong Kong Baptist University, Kowloon Tong, Hong Kong SAR; 2 HKBU Institute of Research and Continuing Education, Shenzhen Virtual University Park, Shenzhen, China; 3 Key Laboratory of Public Health Safety, Ministry of Education, School of Public Health, Fudan University, Shanghai, 200032 China; 4 Key Lab of Health Technology Assessment, National Health Commission of the People’s Republic of China (Fudan University), Shanghai, China

**Keywords:** Chemistry, Endocrine system and metabolic diseases

## Abstract

The aim of this pilot study was to predict the risk of gestational diabetes mellitus (GDM) by the elemental content in fingernails and urine with machine learning analysis. Sixty seven pregnant women (34 control and 33 GDM patient) were included. Fingernails and urine were collected in the first and second trimesters, respectively. The concentrations of elements were determined by inductively coupled plasma-mass spectrometry. Logistic regression model was applied to estimate the adjusted odd ratios and 95% confidence intervals. The predictive performances of multiple machine learning algorithms were evaluated, and an ensemble model was built to predict the risk for GDM based on the elemental contents in the fingernails. Beryllium, selenium, tin and copper were positively associated with the risk of GDM while nickel and mercury showed opposite result. The trained ensemble model showed larger area under curve (AUC) of receiver operating characteristic curve (0.81) using fingernail Ni, Cu and Se concentrations. The model was validated by external data set with AUC = 0.71. In summary, the results of the present study highlight the potential of fingernails, as an alternative sample, together with machine learning in human biomonitoring studies.

## Introduction

Gestational diabetes mellitus (GDM) is one of the most common pregnancy complications threatening both maternal and fetal health^1^. The prevalence of GDM in China is 14.8%, which is the largest worldwide^2^. GDM is known as impaired glucose tolerance during pregnancy. In healthy pregnant women, the demand of insulin increases to store glucose for later stages of pregnancy. However, the dysfunction of pancreatic β-cell occurred in GDM pregnant women resulted in insufficient of insulin and causes hyperglycemia^3^. In literature, there is increasing evidence that certain heavy metals in pregnant women is associated with the risk of GDM. For example, several meta-analyses have reported that increased levels of arsenic (As)^4^, iron (Fe)^5^ and cadmium (Cd)^6^ were associated with the risk of GDM. However, contradictory results have been reported. For instance, two recently published studies evaluated the association between multi-elements and GDM. One of them reported that urinary Ni was positively associated with GDM^7^ while another reported no significant association between Ni and GDM^8^. More studies are required for confirming an association between heavy metals and GDM.

Studies involving nail samples (i.e., fingernails, toenails) in human biomonitoring (HBM) have proliferated in recent years due to the ease of collection and their biological properties compared with blood and urine^9^. The major advantages of nails over blood and urine are that samples represent long-term accumulation; collection is simple, easy and non-invasive; and storage and transport are also simple, easy, and convenient. Due to the slow growth rate of nails, nails record exposure over periods ranging from a few weeks to more than a year^10^. The correlation between elemental contents in nail samples and diabetes have been evaluated in various studies. Copper (Cu)^11^ and selenium (Se)^12^ were inversely associated with the risks of diabetes and obesity. In contrast, exposure to mercury (Hg) and nickel (Ni)^13^ increased the risk of diabetes. There has been only one study, however, assessing the relationship between elements in nails and GDM. That study evaluated the correlation between As in toenails and the risk of GDM^14^. Understanding the correlation between nail elements and GDM may promote the use of nails as a simple, non-invasive way of monitoring the risk for GDM in clinical applications.

Machine learning analysis is an emerging trend in the field of HBM. Conventional statistical models describe the features of data based on various assumptions rather than predicting the risk of disease development. In contrast, machine learning aims at developing models through general learning algorithms from data to predict outcomes^15^. Machine learning has been widely applied for predicting and/or classifying different diseases based on elemental contents in the body. The concentration of six elements in cerebrospinal fluid was used to predict the risk of Parkinson’s disease using Support Vector Machine Model^16^. In another study, healthy individual and nasopharyngeal carcinoma (NPC) patients were accurately classified according to the elemental contents in their blood serum. This study highlights the potential of early diagnosis of NPC. In terms of GDM prediction by machine learning, several biomarkers in blood have provided acceptable prediction for the risk of GDM^17–19^. The many known risk factors of GDM, such as age, pre-pregnancy BMI and family history of diabetes^20^, have also been proved to be valuable in the prediction of GDM using machine learning^20–22^. However, these risk factors failed to fully elucidate the etiology of GDM. As mentioned previously, elements were associated with the risk of GDM. Machine learning is a good way to explore and establish the predictive value of elements together with conventional risk factors for the incidence of GDM. Moreover, fingernail samples used in this study reflected elemental contents in pregnant women well before the onset of GDM, demonstrating a great potential for early prediction on the risk of GDM. To the best of our knowledge, this is the first study applying machine learning for GDM prediction based on the elemental contents determined in fingernail samples.

The present nested case–control study aimed to demonstrate the ability of machine learning to predict GDM based on analysis of elements in fingernails. Twenty-seven elements were monitored in fingernails by ICP-MS after acid digestion. The risk of GDM was predicted by ensemble subspace model using the elemental contents in fingernails as well as the clinical information. The performance of optimized prediction model trained by fingernails elemental contents was also evaluated.

## Results

### Basic characteristics

The basic characteristics of control and GDM pregnant women are listed in Table 1. No significant associations were observed for the characteristics assessed between control and GDM group. One outlier was observed from the GDM patient fingernail elemental contents while four patient urinary elemental contents were found missing. Hence, only 33 and 30 GDM patients were included in the fingernails and urine statistical analyses, respectively.

**Table 1:** Basic characteristics of control and GDM pregnant women.

Characteristic	Total (n = 67)	Control (n = 34)	GDM (n = 33)	*p* value
Education level				0.33
Junior high school	10 (14.9%)	4 (12.1%)	6 (17.6%)	
High school	12 (17.9%)	7 (21.2%)	5 (14.7%)	
Junior college	20 (29.9%)	7 (21.2%)	13 (38.2%)	
University/above	25 (37.3%)	15 (45.5%)	10 (29.4%)	
Income (CNY per year)				0.85
< 100,000	19 (28.4%)	8 (24.2%)	11 (32.4%)	
100,000–200,000	32 (47.8)	18 (54.5%)	14 (41.2%)	
200,000–300,000	8 (11.9%)	3 (9.1%)	5 (14.7%)	
300,000–400,000	4 (6.0%)	2 (6.1%)	2 (5.9%)	
400,000–500,000	4 (6.0%)	2 (6.1%)	2 (5.9%)	
Passive smoking				0.39
Yes	16 (23.9%)	6 (18.2%)	10 (29.4%)	
No	51 (76.1%)	27 (81.8%)	24 (70.6%)	
Physical activity pattern				0.55
Low strength	28 (41.8%)	13 (39.4%)	15 (44.1%)	
Middle strength	37 (55.2%)	18 (54.5%)	19 (55.9%)	
High strength	2 (3.0%)	2 (6.1%)	0 (0.0%)	
Family history of diabetes				1.00
Yes	65 (97.0%)	32 (97.0%)	33 (97.1%)	
No	2 (3.0%)	1 (3.0%)	1 (2.9%)	
Parity				0.63
1 time	34 (50.7%)	18 (54.5%)	16 (47.1%)	
> 1 time	33 (49.3%)	15 (45.5%)	18 (52.9%)	
Age				
Mean (SD)	30.7 (± 4.3)	30.9 (± 4.1)	30.5 (± 4.5)	0.65
Pre-pregnancy BMI				
Mean (SD)	21.9 (± 3.5)	22.2 (± 3.7)	21.6 (± 3.3)	0.29

*p* value of age and pre-pregnancy BMI were calculated by Mann–Whitney *U* test;

*p* value of education level, income, passive smoking, physical activity pattern, family history of diabetes and parity were calculated by Pearson Chi-square test.

### Elemental contents in fingernails

The detection rates, median concentrations, and the interquartile ranges (IQR) of elements in fingernails are summarized in Table 2. The detection rates of beryllium (Be), arsenic (As), molybdenum (Mo), cerium (Ce) and mercury (Hg) were below 90%. The concentrations of Be (*p* value < 0.001), selenium (Se) (*p* value = 0.003), tin (Sn) (*p* value = 0.009) and antimony (Sb) (*p* value = 0.032) in fingernails of GDM patient were significantly higher than those of the control group while the concentration of nickel (Ni) (*p* value = 0.029) and mercury (Hg) (*p* value = 0.005) in fingernails of GDM patients showed the opposite trend.

**Table 2:** Elemental concentrations in fingernails of control and GDM group.

Elements	Below LOD (%) (n = 67)	Control (n = 34)	GDM (n = 33)	*p* value
Li	0 (0.0%)	16.13 (12.70–27.11)	15.97 (12.67–20.46)	0.866
Be	**27 (40.3%)**	** < LOD (< LOD-0.18)**	**0.16 (0.09–0.74)**	** < 0.001**
Mg*	0 (0.0%)	90.02 (77.40–96.87)	83.17 (72.88–96.29)	0.807
Al*	0 (0.0%)	19.16 (13.74–30.19)	21.09 (15.35–27.85)	0.603
V	0 (0.0%)	40.26 (28.83–58.33)	41.19 (26.46–65.88)	1
Cr	0 (0.0%)	292.10 (184.39–511.32)	330.30 (219.68–489.79)	0.730
Mn	0 (0.0%)	340.44 (224.68–571.81)	321.89 (265.13–500.33)	0.885
Fe*	0 (0.0%)	26.54 (21.53–33.47)	26.21 (20.03–33.51)	0.577
Co	0 (0.0%)	16.14 (11.89–23.99)	17.53 (13.35–30.27)	0.377
Ni	**0 (0.0%)**	**793.81 (505.64–1603.92)**	**409.46 (313.36–890.30)**	**0.029**
Cu	0 (0.0%)	3972.89 (3186.18–5258.24)	4398.95 (3798.94–5369.89)	0.278
Zn*	0 (0.0%)	81.42 (76.63–87.00)	87.23 (76.33–96.86)	0.121
As	37 (55.2%)	3.30 (< LOD-43.76)	< LOD (< LOD-21.65)	0.306
Se	**0 (0.0%)**	**461.54 (400.94–543.12)**	**532.95 (481.76–665.16)**	**0.003**
Sr	0 (0.0%)	770.16 (443.18–1065.63)	756.93 (492.83–1093.87)	1
Mo	13 (19.4%)	5.95 (1.00–17.79)	4.61 (0.45–18.67)	0.826
Cd	0 (0.0%)	23.91 (16.75–57.42)	21.26 (11.09–32.90)	0.158
Sn	**0 (0.0%)**	**315.44 (199.85–576.30)**	**561.75 (295.81–733.55)**	**0.009**
Sb	**0 (0.0%)**	**39.27 (27.95–57.71)**	**49.02 (35.15–88.80)**	**0.032**
Ba	0 (0.0%)	867.26 (573.90–1375.13)	752.44 (552.27–1283.23)	0.551
La	0 (0.0%)	10.98 (8.28–16.54)	11.47 (7.67–17.02)	0.945
Ce	17 (25.4%)	7.62 (< LOD-21.87)	7.84 (0.99–16.38)	0.884
Hg	**11 (16.4%)**	**92.09 (44.09–141.39)**	**32.43 (0.02–76.56)**	**0.005**
Tl	0 (0.0%)	0.37 (0.20–0.47)	0.38 (0.26–0.59)	0.307
Pb	0 (0.0%)	617.40 (277.10–1073.17)	560.91 (364.66–865.72)	0.846
Bi	4 (6.0%)	5.34 (2.04–12.38)	4.67 (2.83–10.90)	0.812
U	0 (0.0%)	3.79 (2.78–6.30)	4.99 (2.63–9.35)	0.246

Concentration presented in median (IQR) (ng/g).

*Concentration presented in median (IQR) (µg/g).

Bolded: *p* value calculated by Mann–Whitney *U* test.

### Associations between elemental concentration in fingernails and GDM

The association between the elemental concentrations in fingernails and GDM was calculated by logistic regression model. Table 3 lists the crude odd ratio with the 95% confidence interval of each element. Among the 27 elements, Se (OR: 43.49, 95% CI 3.54–847.67), Sn (OR: 2.32, 95% CI 1.13–5.55) and Be (OR: 1.52, 95% CI 1.23–1.97) were positively association with GDM. Ni (OR: 0.50, 95% CI 0.25–0.92) and Hg (OR: 0.73, 95% CI 0.58–0.89) were negatively associated with GDM. We further analyzed the association between elements and the risk of GDM based on the tertiles of elemental concentrations (Table 4). The risk of GDM increased with the concentrations of Be (OR: 8.64, 95% CI 2.04–45.44 in the highest tertile), Cu (OR: 8.08. 95% CI 1.93–41.78 in the second tertile), Se (OR: 4.67, 95% CI 1.23–19.73 in the highest tertile) and Sn (OR: 6.78, 95% CI 1.68–32.34 in the highest tertile). Significant positive dose–response relationships were observed for Be (adjusted *p* for trend: 0.090) and Sn (adjusted *p* for trend: 0.090). The risk of GDM decreased with increased concentrations of Ni (OR: 0.020, 95% CI 0.05–0.77 in the highest tertile) and Hg (OR: 0.21, 95% CI 0.05–0.84 in the second tertile and OR: 0.10, 95% CI 0.02–0.41 in the highest tertile). Significant negative dose–response relationship was observed for Hg (adjusted *p* for trend: 0.081).

**Table 3:** Adjusted odd ratio (OR) of elements in fingernails for the risk of GDM.

Element	OR (95%CI)	*p* value	Adjusted *p* value	Element	OR (95%CI)	*p* value	Adjusted *p* value
Li	0.71 (0.29, 1.52)	0.405	0.810	Sr	1.03 (0.43, 2.51)	0.943	0.943
Be	**1.52 (1.23, 1.97)**	**0.000**	**0.000**	Mo	0.99 (0.88, 1.11)	0.858	0.918
Mg	1.67 (0.16, 20.87)	0.676	0.810	Cd	0.56 (0.25, 1.15)	0.421	0.810
Al	1.32 (0.51, 3.46)	0.569	0.810	Sn	**2.32 (1.13, 5.55)**	**0.037**	**0.077**
V	1.22 (0.44, 3.50)	0.703	0.810	Sb	2.11 (0.99, 5.31)	0.076	0.200
Cr	0.84 (0.34, 1.99)	0.683	0.810	Ba	0.87 (0.38, 1.91)	0.720	0.810
Mn	1.25 (0.48, 3.37)	0.650	0.810	La	0.95 (0.47, 1.84)	0.884	0.918
Fe	0.71 (0.16, 2.99)	0.647	0.810	Ce	1.03 (0.94, 1.13)	0.530	0.810
Co	1.28 (0.51, 3.34)	0.592	0.810	Hg	**0.73 (0.58, 0.89)**	**0.003**	**0.041**
Ni	**0.50 (0.25, 0.92)**	**0.035**	**0.077**	Tl	1.64 (0.62, 4.56)	0.322	0.810
Cu	4.26 (0.80, 26.80)	0.101	0.342	Pb	1.15 (0.59, 2.30)	0.679	0.810
Zn	5.43 (0.40, 141.57)	0.262	0.390	Bi	1.08 (0.93, 1.28)	0.332	0.810
As	0.95 (0.84, 1.07)	0.421	0.810	U	1.42 (0.78, 2.75)	0.272	0.810
Se	**43.49 (3.54, 847.67)**	**0.006**	**0.041**				

Odd ratio adjusted for education level, income, passive smoking, physical activity pattern, family history of diabetes, parity, age and pre-pregnancy BMI.

Bold: *p* value < 0.05 by logistic regression analysis.

FDR correction was indicated in adjusted *p* value (significant level: *p* value < 0.1).

**Table 4:** Adjusted odd ratio (OR) for the risk of GDM according to the tertiles of fingernail elemental concentration.

Element	Tertile	OR (95%CI)	*p* value	Element	Tertile	OR (95%CI)	*p* value
Li	Q1	ref	ref	Cu	Q1	ref	ref
	Q2	0.48 (0.13, 1.75)	0.274		Q2	**8.08 (1.93, 41.78)**	**0.007**
	Q3	0.90 (0.26, 3.11)	0.863		Q3	2.44 (0.65, 9.86)	0.193
***P for trend***			0.945	***P for trend***			0.624
Be	Q1	ref	ref	Zn	Q1	ref	ref
	Q2	2.78 (0.72, 11.85)	0.147		Q2	0.30 (0.07, 1.23)	0.106
	Q3	**8.64 (2.04, 45.44)**	**0.006**		Q3	2.61 (0.68, 10.74)	0.169
***P for trend***			**0.090**	***P for trend***			0.493
Mg	Q1	ref	ref	As	Q1	ref	ref
	Q2	0.68 (0.17, 2.63)	0.581		Q2	2.18 (0.61, 8.24)	0.235
	Q3	0.91 (0.24, 3.48)	0.884		Q3	1.03 (0.27, 4.02)	0.963
***P for trend***			0.945	***P for trend***			0.945
Al	Q1	ref	ref	Se	Q1	ref	ref
	Q2	1.27 (0.34, 4.81)	0.720		Q2	3.00 (0.82, 11.93)	0.103
	Q3	1.61 (0.46, 5.82)	0.457		Q3	**4.67 (1.23, 19.73)**	**0.028**
***P for trend***			0.945	***P for trend***			0.140
V	Q1	ref	ref	Sr	Q1	ref	ref
	Q2	0.87 (0.25, 3.01)	0.822		Q2	1.13 (0.31, 4.22)	0.849
	Q3	1.38 (0.36, 5.51)	0.644		Q3	1.07 (0.27, 4.30)	0.925
***P for trend***			0.945	***P for trend***			0.945
Cr	Q1	ref	ref	Mo	Q1	ref	ref
	Q2	1.52 (0.42, 5.74)	0.524		Q2	1.05 (0.29, 3.77)	0.944
	Q3	1.35 (0.32, 5.88)	0.679		Q3	1.17 (0.31, 4.46)	0.816
***P for trend***			0.945	***P for trend***			0.945
Mn	Q1	ref	ref	Cd	Q1	ref	ref
	Q2	1.55 (0.45, 5.58)	0.492		Q2	0.27 (0.06, 1.00)	0.056
	Q3	1.34 (0.37, 5.02)	0.657		Q3	0.32 (0.07, 1.39)	0.135
***P for trend***			0.945	***P for trend***			0.493
Fe	Q1	ref	ref	Sn	Q1	ref	ref
	Q2	1.01 (0.27, 3.80)	0.985		Q2	2.38 (0.62, 9.88)	0.215
	Q3	1.09 (0.29, 4.20)	0.896		Q3	**6.78 (1.68, 32.34)**	**0.010**
***P for trend***			0.945	***P for trend***			**0.090**
Co	Q1	ref	ref	Sb	Q1	ref	ref
	Q2	2.62 (0.65, 11.71)	0.186		Q2	0.94 (0.25, 3.53)	0.930
	Q3	1.24 (0.34, 4.53)	0.746		Q3	2.46 (0.68, 9.67)	0.179
***P for trend***			0.945		***P for trend***		0.493
Ni	Q1	ref	ref	Ba	Q1	ref	ref
	Q2	0.38 (0.10, 1.39)	0.152		Q2	0.76 (0.21, 2.68)	0.669
	Q3	**0.20 (0.05, 0.77)**	**0.024**		Q3	0.74 (0.19, 2.90)	0.660
***P for trend***			0.140	***P for trend***			0.945
La	Q1	ref	ref	Pb	Q1	ref	ref
	Q2	1.06 (0.29, 3.93)	0.928		Q2	1.57 (0.44, 5.78)	0.489
	Q3	1.06 (0.30, 3.77)	0.930		Q3	1.15 (0.28, 4.81)	0.845
***P for trend***			0.945	***P for trend***			0.945
Ce	Q1	ref	ref	Bi	Q1	ref	ref
	Q2	2.46 (0.65, 10.08)	0.194		Q2	2.88 (0.76, 11.89)	0.127
	Q3	1.01 (0.27, 3.82)	0.989		Q3	0.98 (0.28, 3.44)	0.972
***P for trend***			0.945	***P for trend***			0.945
Hg	Q1	ref	ref	U	Q1	ref	ref
	Q2	**0.21 (0.05, 0.84)**	**0.034**		Q2	0.76 (0.20, 2.83)	0.685
	Q3	**0.10 (0.02, 0.41)**	**0.003**		Q3	2.28 (0.66, 8.28)	0.199
***P for trend***			**0.081**	***P for trend***			0.606
Tl	Q1	ref	ref				
	Q2	0.80 (0.21, 2.93)	0.735				
	Q3	1.37 (0.36, 5.27)	0.639				
***P for trend***			0.945				

Odd ratio adjusted for education level, income, passive smoking, physical activity pattern, family history of diabetes, parity, age and pre-pregnancy BMI.

ref: reference group.

*p* for trend was adjusted by FDR correction.

Bold indicates p value < 0.05 by logistic regression analysis.

### Prediction performance of ensemble model

Machine learning analysis can help predict diseases including GDM. In the present study, we utilized machine learning to evaluate the correlation between multi-element contents and the risk of GDM based on the results from the traditional statistical analysis. According to the logistic regression analysis shown above, Be, Ni, Cu, Se, Sn and Hg were found significantly associated with the risk of GDM. Due to the low detection rate of Be and Hg, they were not included in the machine learning algorithm to minimize the bias generated by the accumulation of larger portion of data points.

The training data set was firstly trained by SVM, KNN, DA, ensemble and NB to select the most accurate algorithm for further analysis. The performances of trained models were compared using the AUC of ROC. The average AUC of the ensemble subspace algorithm resulted in the highest AUC (0.78) with the smallest standard deviation among the tested algorithms. Because this indicate a higher reproducibility, it was used in further analysis (Supplementary Fig. S1).

In the first attempt, a single element was used to train models; the results are shown in Supplementary Table S5. In order to further enhance the accuracy of the trained models, multi-element models were employed to improve the prediction performance. All possible combinations were evaluated by the ensemble model with the best results summarized in Table 5. The highest AUC obtained was 0.81 using Ni, Cu and Se as predictors. When Sn was added to the trained model, the AUC did not change significantly while the accuracy and sensitivity of models decreased significantly to 0.65 and 0.71 respectively. Hence, the combination of Ni, Cu and Se was evaluated together with the basic characteristics of participants to maximize the prediction accuracy. Finally, six different basic characteristics were added on top of the combination of Ni, Cu and Se to evaluate the prediction performance. However, the prediction performance of trained models deteriorated due to the decrease of sensitivity (Supplementary Table S6). Hence, models trained by Ni, Cu and Se without any basic characteristics were further validated by testing data set in the subsequent study.

**Table 5:** Prediction performance of multi-element model trained by fingernail elemental contents.

No. of element in group	Element	AUC	Sensitivity	Accuracy	Balanced accuracy	F-value	Matthews correlation coefficient	*p* value of permutation test
4	Ni, Cu, Se, Sn	0.80	0.65	0.71	0.66	0.60	0.33	0.02
3	Ni, Cu, Se	0.81	0.76	0.78	0.78	0.78	0.57	< 0.01
2	Ni, Cu	0.78	0.53	0.68	0.67	0.62	0.36	0.02
1	Cu	0.73	0.72	0.73	0.73	0.72	0.45	0.02

AUC, area under the receiver operating characteristic curve.

No. of permutations: 100.

### Comparison of prediction performance of models constructed from fingernails and urine data

The association of urinary elements with GDM and the prediction performance with urinary elements are detailed in Supplementary Table S3. Among the 10 elements (Li, Mg, Ni, Cu, Zn, As, Se, Sr, Mo, Sn) with detection rates at 90% or above, none showed significant association between control and GDM groups. There was also lack of significant dose–response relationships between the target elements and the risk of GDM (Supplementary Table S8).

**Figure 1:**
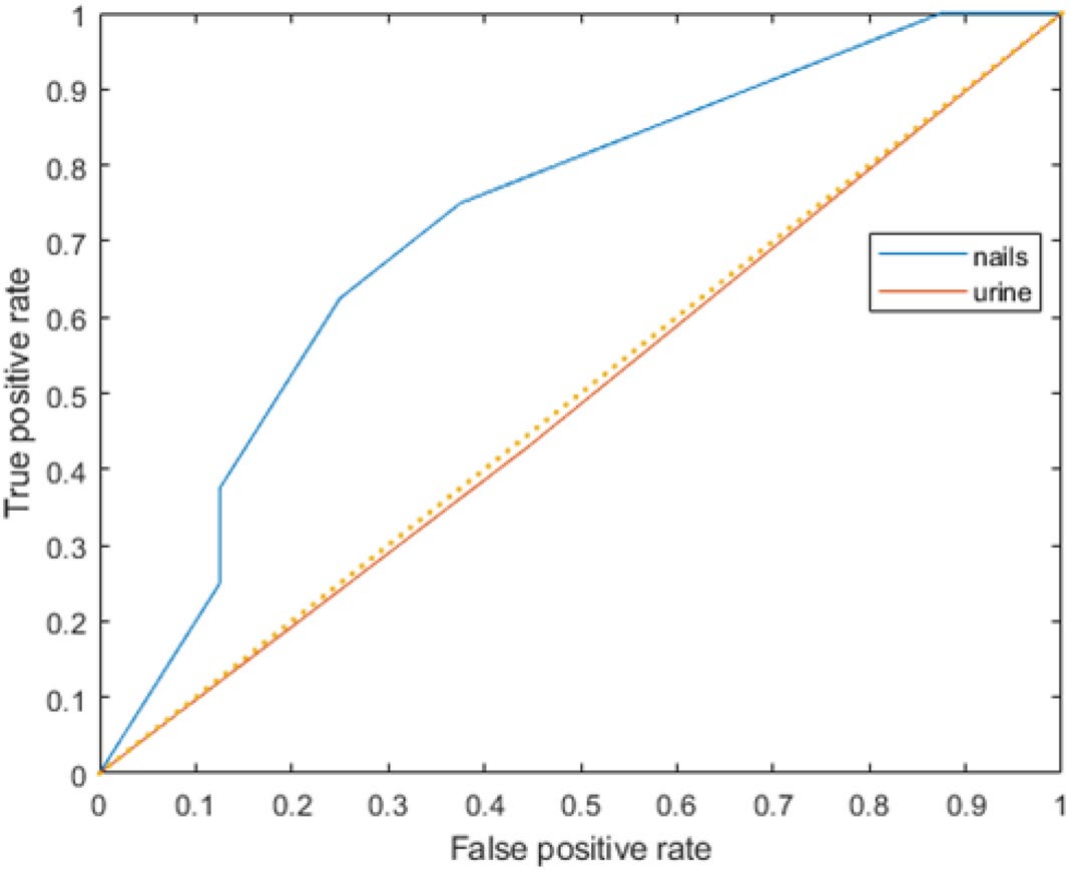
ROC curve of best predictions given by nails and urine with ensemble model and KNN model respectively. The prediction performance of trained models were validated by testing data set. The AUC of fingernail and urine prediction models were 0.71 and 0.49, respectively.

The machine learning analysis of urinary elements was carried out like fingernails. As shown in Supplementary Fig. S2, kNN and ensemble models resulted in similar AUCs. The training process of kNN was much faster than that of the ensemble model; hence the kNN model was used for training models with urinary elemental contents. The best prediction combination was given by urinary Cu, Se and Sn concentrations in addition to pre-pregnancy BMI, physical activity pattern and parity (Supplementary Table S11). After selecting the optimized models for fingernails and urine respectively, the testing data set was used to validate the prediction performance of both models. Figure 1 shows that the trained fingernail model (AUC: 0.71) performed better than the urine model (AUC: 0.49) in predicting GDM.

## Discussion

This is the first study predicting the risk of GDM based on the elemental content of fingernails using a machine learning algorithm. A similar approach has been used to evaluate the risk of GDM based on the metabolites of urine^23^. Conventional statistical models have been widely applied to evaluate the association between elements and GDM in many studies^14,24,25^. However, no studies have applied machine learning for this purpose. In the present study, we first used conventional statistical models, and found significant associations of Be, Ni, Se, Sn, Sb, Cu and Hg with GDM (Table 2 to Table 4). We here present the first report of a significant association between Be concentration and GDM. Nevertheless, statistical models cannot conclusively determine the risk of GDM solely by the association with elements. According to Senat, et al., many other basic characteristics including age, pre-pregnancy BMI, and family history of diabetes are general risk factors for GDM^26^. Passive smoking^24^; parity^25^ has also been reported as a potential risk factor. Machine learning can take into account these many factors. Hence, machine learning analysis was implemented to find the hidden pattern in multi-factorial data collected from pregnant women with and without GDM, and then predict the risk of GDM with trained models.

Numerous machine learning models for the prediction of GDM have been reported^27,28^; however, there is no consensus as to which one is best. As shown in Supplementary Fig. S1, the prediction performances of 15 machine learning algorithms were compared using the training data set. Ensemble models and SVM models resulted in similar AUC in the trained models, but the ensemble subspace model was more reproducible, suggesting it would be more reliable for the data in the present study. The major advantage of ensemble models over SVM is that ensemble models use multiple single models to form a new model. As a result, the prediction performance of an ensemble algorithm is usually better than a single algorithm^29^. After selecting the algorithms, different combinations of elements as well as basic characteristics were used to train models to obtain the highest accuracy.

The model was firstly trained by single element content in fingernails. The results of single element models (Supplementary Table S5) show that only the ensemble model trained by Cu level in fingernails provided acceptable prediction performance. Multiple studies have reported that multi-elements exposure is significantly associated with GDM^7,8^. Hence, we also evaluated the performance of models trained by multiple elements, ranging from two element combinations to four element combinations. Table 5 shows that when the number of elements increased, the prediction performance of the trained model also increased. The trained model was validated by an external testing data set. Figure 1 shows that acceptable accuracy was obtained by the trained model, which suggested that the concentrations of Cu, Ni and Se were important predictors for GDM. In the present study, addition of the basic characteristics of pregnant women did not improve the prediction performance of the machine learning models. It indicated that the models used in the present study worked better for numerical variables but not categorical variables^30^.

The elements used to train the predictive model were similar to most of the other studies. The correlation between circulating Cu level and GDM was summarized using the data from 14 published studies. It was concluded that high serum Cu was positively associated with the risk of GDM, especially among Asians during the third trimester^31^. Multiple systematic reviews and meta-analyses have focused on the association between Se and GDM. Those studies were consistent in concluding that Se concentrations were low in women with GDM compared with normal women, while the present study shows an opposite trend. The studies involved in those reviews determined serum Se level in either second or third trimesters^32–34^; while in this study Se levels were measured in the first trimester. Studies reporting the correlation of blood or urinary Ni with GDM are limited, and the conclusions are inconsistent. The present study found significant negative association between fingernail Ni level and GDM while the above mentioned studies reported no significant association^8^ and positive association^7^, respectively. Our results show that the correlation between fingernail elements and GDM is different from that of blood and urine.

Although the trained model in the present study did not include basic characteristic as predictors, our models highlighted fingernail Cu, Ni and Se concentrations as potential predictors for GDM. To the best of our knowledge, this is the first study demonstrating the prediction of GDM by elemental contents using machine learning. Our model outperformed the models trained by serum triglyceride and fasting plasma glucose level (AUC: 0.68)^17^. Our trained model also performed comparably to another model trained by cytosine-phosphate-guanine levels in blood (AUC: 0.82)^19^. Although excellent prediction models constructed by putrescine and microRNA with AUC 0.95 and 0.91, respectively, have been reported, studies using those models did not include external validation by a testing data set^18,27^. Our prediction model was validated by a testing data set and resulted in AUC 0.71, which indicated acceptable performance.

Another major highlight of the present study is that fingernail samples were collected in the first trimester. To date, many studies involving nail samples utilized nail clippings collected either during a later stage of pregnancy or postpartum^35^. Information obtained from nail samples represents exposure from a few weeks to a few months before collection^36^. As a result, the association observed using those samples is closely related to the middle to later stage of pregnancy. In contrast, the fingernail samples used in this study represent exposure during the first few weeks of gestation, if not before, which is much earlier than the identification of GDM. But this is what prediction means: Anticipating a problem before it develops. The model used in this pilot study highlights the ability of fingernail Cu, Ni and Se levels to predict GDM because it was predicting the risk of GDM before the development of GDM.

In the present work, we collected both urine and fingernail samples from the same individual and predicted the risk of GDM with their elemental contents through machine learning analysis. One of the major advantages of using fingernails rather than urine is that the elemental detection rate in fingernails is higher than that in urine. The elemental analysis revealed more than 90% of 24 elements in fingernail samples, while the same analysis could detect only 9 elements in urine samples. For fingernails, it should be pointed out that although the detection rates of Be and Hg were relatively low, our model found that they had a significant association with the risk of GDM. In terms of the prediction performance of the trained model, prediction by fingernail elemental contents provided acceptable predictive accuracy for the testing data set while the prediction by urinary elemental contents was similar to random guessing, as the AUC was 0.49 for the external validation result of a urine prediction model (Fig. 1)^37^. It was mainly due to the low elemental detection rate and no significant difference in elemental concentrations between control and GDM patients for urine sample (Supplementary Table S3). Although it is expected that the use of urine sample will remain dominant in HBM studies, this pilot study highlights that fingernails are a potential alternative sample for predicting the risk of GDM.

However, there are several important limitations that should be considered in interpreting the results of the present study. Firstly, the sample size was relatively small. A larger sample (more than 1000 pregnant women in total) will be utilized in the future study to compare the prediction performance of models with other studies^38^. Secondly, the reason why the results of this study with regard to the correlation between some of the elements with GDM were not consistent with past studies is not known. For example, As content in urine or blood is well-known for its correlation with GDM but no significant association was observed in the present study^39,40^. To date, there is only one study reported As content in toenails in association with GDM, and it found that As content in toenails collected 2 weeks postpartum was significantly associated with GDM^14^. Our study utilized fingernails, collected in the first trimester. The influence of type of nails and the specific stage of pregnancy needs to be thoroughly examined in future studies, and other reasons for these inconsistencies need to be explored. Thirdly, the urinary elemental detection rates in the present study were low, which affected the results of machine learning.

## Conclusion

To the best of our knowledge, this is the first study demonstrating the application of machine learning analysis to the prediction of GDM using the elemental contents in fingernails. Our study provides additional evidence for the positive association between elemental contents in fingernails and GDM. The results indicate that Ni, Cu and Se concentrations, in particular, in fingernails are important factors for the prediction of GDM by ensemble subspace models. In contrast with fingernails, the elemental contents in urine failed to predict the risk of GDM due to the low detection rate for most of the elements. The present study highlights the potential of GDM prediction in early pregnancy using the elemental contents in fingernails. Further large scale studies are required to verify the correlation of elemental contents in fingernails and GDM. It should be pointed out that long-term exposure information provided by fingernails may also help in elucidating the mechanistic relationship between elements and GDM development.

## Methods

### Study population

This pilot, nested case–control study was based on the Shanghai Maternal-Child Pairs cohort study conducted at the School of Public Health, Fudan University, Shanghai. A cohort of Shanghai pregnant women were recruited from September 2016 to December 2017. Eligible women were those who: (1) were over 20 years old; (2) were free of serious chronic disorders (e.g., diabetes, high blood pressure, heart disease, etc.); (3) did not smoke or drink alcohol. Of these, 34 with GDM were recruited for the evaluation of the association between elemental exposure and the risk of GDM. Diagnosis of GDM was performed by oral glucose tolerance test (OGTT) during gestational weeks 24–28. 34 pregnant women without GDM were selected as control group by propensity score matching. The control group was matched with the experimental group in terms of age, living district, pre-pregnancy BMI, family yearly income, education level, infant sex, parity, passive smoking, and physical activity pattern.

### Ethic statement

All methods were carried out in accordance with relevant guidelines and regulations. The study was approved by the Institutional Review Boards (IRB) of the School of Public Health, Fudan University (IRB#2016-04-0587). Informed consent was obtained for each participant at the time of enrollment.

### Data collection

A face-to-face interview was conducted by a trained nurse with each participant using a standard questionnaire to collect information on age, pre-pregnancy BMI, education level, family yearly income, passive smoking, physical activity pattern, parity and family history of diabetes. Information on the oral glucose tolerance test (OGTT) of pregnant women, and infant sex were retrieved from medical records.

### GDM diagnosis

The diagnosis of GDM was based on the guideline published by the Ministry of Health (MOH) of China^41^. A 75 g OGTT was performed on pregnant women during gestational weeks 24–28. Pregnant women were identified as having GDM if any one of the following criteria was met: fasting plasma glucose ≥ 5.1 mmol/L, 1 h plasma glucose ≥ 10.0 mmol/L or 2 h plasma glucose ≥ 5.1 mmol/L.

### Sample collection

Fingernail samples (width larger than 0.2 mm) were provided by pregnant women during 12–16 weeks of gestation. They were asked to collect nails from all fingers with a stainless-steel nail clipper. Fingernail samples were stored in a zip-locked plastic bag at room temperature and transported to Hong Kong Baptist University (HKBU) for elemental analysis. For each individual, the mass of fingernail samples used for ICP-MS analysis ranged from 5 to 10 mg.

Urine samples (10 mL) were collected at around 16 weeks of gestation. Samples were stored in polypropylene centrifuge tubes at -80 °C until analysis.

### Elemental analysis

The concentration of 27 elements in fingernails was determined by inductively coupled plasma-mass spectrometry (ICP-MS) at HKBU while the concentration of the same 27 elements in urine was determined by ICP-MS in Shanghai. Fingernail samples were washed using the method recommended by the International Atomic Energy Agency (IAEA)^42^. The subsequent acid-digestion was performed using a modified method reported in our previous study^43^. In brief, fingernail samples were transferred to 15 mL polypropylene tubes and then rinsed by acetone followed by ultrapure water three times. The washed samples were oven-dried at 60 °C overnight. Samples were then mineralized with 3 mL of concentrated nitric acid and 1 mL of 30% hydrogen peroxide in a microwave digestion system. The digested solution was transferred to an acid-washed beaker and evaporated on a hotplate until nearly dry. 5 µL of 1 µg/mL germanium standard solution was added as an internal standard and ultrapure water was used to make up the sample solution to 5 mL. The solution was then analyzed by ICP-MS. Multi-element standard solutions (Li, Be, Mg, V, Cr, Mn, Ni, As, Se, Mo, Cd, Sn, Sb, La, Ce, Hg, U: 0.2–10 ng/mL; Co, Tl, Bi: 0.04–2 ng/mL; Sr, Pb: 0.8–40 ng/mL; Cu, Ba: 2–100 ng/mL; Fe: 4–200 ng/mL; Al-Zn: 8–400 ng/mL) were prepared by appropriate dilution of 1000 µg/mL of stock standard solution for the quantification of elements by external calibration. Since certified reference material (CRM) for nails was not available, hair CRM was employed for method validation. 5 mg of hair CRM or fingernail sample was digested and analyzed by ICP-MS. The standard solution used in the calibration was analyzed every 20 samples to ensure no significant instrumental drift occurred during the analysis.

Urine samples were thawed at room temperature and vortexed (IKA, Germany) for 5 s. Then each sample was centrifuged (Microfuge 16, Beckman Coulter, USA) at 4000 rpm for 1 min to remove debris. 1 mL of supernatant was diluted with 1% (*v/v*) concentrated nitric acid (HNO_3_) to 10 mL and vortexed for 30 s to ensure complete mixing. Other relevant details can be found in the Supplementary Materials.

### Statistical analysis

Limit of Detections (LODs) were calculated by multiplying the standard deviation of 7 consecutive measurements of blank standard solution by three, and then dividing that number by the slope of the calibration curve. Calculated concentrations below LOD were assigned the value as LOD/2 for further analysis. Elements with detection rates higher than 90% were included in the machine learning algorithm. The basic characteristics of the participants are summarized in Table 1. The education level, income, passive smoking, physical activity pattern, family history of diabetes and parity were compared by Pearson chi-square test. Mann–Whitney *U* test was used to examine the age and pre-pregnancy BMI. The association between elements and the risk of GDM were evaluated by Mann–Whitney *U* test and logistic regression analysis. Odd ratio (OR) and 95% confidence intervals (CIs) were calculated with elemental concentration as continuous variables and categorical variables according to the tertile distribution (The first tertile was used as the reference group) for the risk of GDM. The regression model was adjusted for age, pre-pregnancy BMI, education level, income, passive smoking, physical activity pattern, family history of diabetes, and parity. The Pearson correlation between fingernail and urinary elemental concentration was determined by MATLAB R2021b software. Unless otherwise specified, a two-tailed *p* value < 0.05 was defined as statistically significant. False discovery rate (FDR) was employed for multiple testing with significant level defined at *p* value < 0.1.

Machine learning analysis was performed by MATLAB R2021b software. Maternal age and pre-pregnancy BMI were input as continuous variables while passive smoking, physical activity pattern, parity and family history of diabetes were input as categorical variables. The prediction accuracy of multiple models, including ensemble models, discriminant analysis (DA), support vector machine (SVM), k-nearest neighbor (kNN) and Naive Bayes (NB), were evaluated. The data was split into a training data set consisting of 51 individuals (75% of total individual) and a testing data set consisting of 16 individuals (25% of total individual, control to patient ratio = 1:1). Ensemble modeling using a random subspace algorithm resulted in the highest area under curve (AUC) in the receiver operating characteristic (ROC) curve for the trained model; these models were used in all analysis in this pilot study. The trained ensemble models were optimized by the training data set with tenfold cross validation repeated five times. The performance of optimized models was evaluated by the test data set. Based on the result of the logistic regression analysis mentioned above, nickel (Ni), copper (Cu), selenium (Se), and tin (Sn) concentrations in fingernails were input as predictors in the ensemble model. Age, pre-pregnancy BMI, passive smoking, physical activity pattern, parity and family history of diabetes were incorporated in machine learning analysis to further optimize the prediction models. Permutation test was employed as the negative control. The *p* value of the permutation test was determined as the fraction obtained by the 100 permutations which were higher than the real accuracy^44^. Balanced accuracy, F-value, Matthews correlation coefficient were also calculated with MATLAB.

## Supplementary Information

Supplementary Information.

## Data Availability

The datasets generated during and/or analysed during the current study are available from the corresponding author on reasonable request.
