# Supplementary material for: A machine learning approach for early prediction of gestational diabetes mellitus using elemental contents in fingernails
Source: Sci Rep. 2023 Mar 14;13:4184. doi: 10.1038/s41598-023-31270-y (PMC10015050; doi:10.1038/s41598-023-31270-y)
Supplement: Supplementary file 1 — Supplementary Information. [file 41598_2023_31270_MOESM1_ESM.docx]

**Supplementary Materials**

**A machine learning approach for early prediction of gestational diabetes mellitus using elemental contents in fingernails**

Yun-Nam Chan^a,1^, Pengpeng Wang^c,d,1^, Ka-Him Chun^a^, Judy Tsz-Shan Lum^a^, Hang Wang^c,d^; Yunhui Zhang^c,d^; Kelvin Sze-Yin Leung^a,b,*^

*^a^ Department of Chemistry, Hong Kong Baptist University, Kowloon Tong, Hong Kong Special Administrative Region*

*^b^ HKBU Institute of Research and Continuing Education, Shenzhen Virtual University Park, Shenzhen, China*

*^c^ Key Laboratory of Public Health Safety, Ministry of Education, School of Public Health, Fudan University, Shanghai 200032, China.*

*^d^ Key Lab of Health Technology Assessment, National Health Commission of the People's Republic of China (Fudan University), China.*

**^*^ Corresponding author:** **Kelvin Sze-Yin Leung,** Department of Chemistry, Hong Kong Baptist University, Kowloon Tong, Hong Kong Special Administrative Region ([s9362284@hkbu.edu.hk](mailto:s9362284@hkbu.edu.hk)).

^1^ Both authors contributed equally to this manuscript

**Content:**

**Reagents and Materials**

**Instrumentation**

**Table S1**. Digestion programme of the microwave system

**Table S2**. Operation parameters of ICP-MS

**Table S3**. Elemental concentrations of urine samples (control and GDM group)

**Table S4**. Spearman correlation of elements between urine and nails

**Table S5**. Prediction performance of single element model trained by fingernail elemental contents

**Table S6**. Prediction performance of multi-element model with basic characteristics trained by fingernail elemental contents

**Table S7**. Adjusted odd ratio (OR) of urinary elements for the risk of GDM

**Table S8**. Adjusted odd ratio (OR) for the risk of GDM according to the tertiles of urinary elemental concentrations

**Table S9**. Prediction performance of single element model trained by urinary elemental contents

**Table S10**. Prediction performance of multi-element model trained by urinary elemental contents

**Table S11**. Prediction performance of multi-element model with basic characteristics trained by urinary elemental contents

**Figure S1**. Comparison on the prediction performance among different trained models by nail elements

**Figure S2**. Comparison on the prediction performance among different trained models by urinary elements

**Reagents and Materials**

Internal standard mixture solutions of Li, Be, Mg, Al, V, Cr, Mn, Fe, Co, Ni, Cu, Zn, As, Se, Sr, Mo, Cd, Sn, Sb, Ba, La, Ce, Hg, Tl, Pb, Bi, U and Ge were prepared by appropriate dilution of respective stock solutions of 1000 mg L^-1^ (High-Purity Standards, Charleston, SC, USA). Milli-Q high purity water (18.2 MΩcm Millipore, Billerica, MA, USA) was used throughout the entire experiment. Hair certified reference material (GBW 07601, Institute of Geophysical and Geochemical Exploration, China) was used to validate the accuracy of elemental analysis for nail samples. Acetone (AR grade) was used to clean nail samples before analysis. Concentrated nitric acid (69%, Aristar, BDH, UK) and hydrogen peroxide (30%, VMR international) were used for acid digestion. All labwares used were immersed in 10% (*v/v*) nitric acid bath for at least 24 h and rinse for three times by ultrapure water before use.

**Instrumentation**

Nail samples were digested in a microwave digestion system (ETHOS-1 Advanced, Milestone S.r.l., Sorisole, Italy) before ICP-MS analysis. The detailed operational parameters for the microwave digestion system are listed in Table S1. Performance of ICP-MS was checked by tuning solution containing 1 ng/L of Li, Co, Y, Ce and Tl everyday before analysis. ICP-MS was optimized with a tuning solution to maximize the sensitivity while minimizing the oxide (CeO/Ce < 1.2%) and the doubly charge (Ce^++^/Ce^+^ < 2.0%) formation. The operation parameters of ICP-MS are listed in the Table S2.

Table S1. Digestion programme of the microwave system

| Step | Time (min) | Temperature (°C) | Power (W) | Pressure (bar) |
| --- | --- | --- | --- | --- |
| 1 | 0 – 3 | 95 | 700 | 25.0 |
| 2 | 3 – 12 | 100 | 1000 | 25.0 |
| 3 | 12 – 37 | 160 | 1000 | 25.0 |
| 4 | 37 – 57 | 180 | 1000 | 25.0 |

Table S2. Operation parameters of ICP-MS

| Parameter | Nails analysis | | Urine analysis |
| --- | --- | --- | --- |
| Instrument | ICP-MS 7900 Agilent | | ICP-MS 7700x Agilent |
| RF power (W) | 1400 | | 1550 |
| Plasma gas flow (L/min) | 14 | | 15 |
| Auxiliary gas (L/min) | 1.0 | | |
| Carrier gas flow (L/min) | 1.05 | | |
| Sample cone | 1.0 mm Nickel | | |
| Skimmer cone | 0.4 mm Nickel | | |
| Sampling depth (mm) | 8 | | |
| Nebulizer and spray chamber | Micromist, Scott-type double-glass (2°C) | | |
| Sample uptake rate (mL/min) | 0.3 | | |
| Detection mode | No gas and He mode | He mode | |
| He gas flow rate (mL/min) | 4.5 | 4.3 | |

Table S3. Elemental concentrations of urine samples (control and GDM group)

| Element | Below LOD (%) (n = 64) | Control (n = 34) | GDM (n = 30) | P-value |
| --- | --- | --- | --- | --- |
| Li | 4 (6.0%) | 13.40 | 10.05 | 0.216 |
| Be | 53 (79.1%) | < LOD | < LOD | 0.676 |
| Mg* | 0 (0.0%) | 81.00 | 68.0 | 0.657 |
| Al* | 42 (62.7%) | < LOD | < LOD | 0.957 |
| V | 66 (98.5%) | < LOD | < LOD | 0.302 |
| Cr | 63 (94.0%) | < LOD | < LOD | 0.673 |
| Mn | 54 (80.6%) | < LOD | < LOD | 0.310 |
| Fe* | 40 (59.7%) | < LOD | < LOD | 0.291 |
| Co | 38 (56.7%) | 0.32 | < LOD | 0.114 |
| Ni | 6 (9.0%) | 2.75 | 2.05 | 0.291 |
| Cu | 0 (0.0%) | 12.90 | 11.90 | 0.633 |
| Zn* | 3 (4.5%) | 221.50 | 253.00 | 0.416 |
| As | 0 (0.0%) | 16.95 | 22.70 | 0.979 |
| Se | 0 (0.0%) | 26.20 | 25.85 | 0.888 |
| Sr | 0 (0.0%) | 119.50 | 125.50 | 0.925 |
| Mo | 0 (0.0%) | 57.50 | 46.00 | 0.231 |
| Cd | 54 (80.6%) | < LOD | < LOD | 0.874 |
| Sn | 8 (11.9%) | 6.70 | 6.65 | 0.793 |
| Sb | 64 (95.5%) | < LOD | < LOD | 0.520 |
| Ba | 40 (59.7%) | < LOD | < LOD | 0.255 |
| La | 67 (100.0%) | < LOD | < LOD | / |
| Ce | 49 (73.1%) | < LOD | < LOD | 0.268 |
| Hg | 65 (97.0%) | < LOD | < LOD | 0.929 |
| Tl | 21 (31.3%) | 0.17 | 0.16 | 0.870 |
| Pb | 29 (43.3%) | 1.02 | 0.70 | 0.363 |
| Bi | 66 (98.5%) | < LOD | < LOD | 0.302 |
| U | 67 (100.0%) | < LOD | < LOD | / |

Concentration presented in median (IQR) (ng/mL)

*Concentration presented in median (IQR) (µg/mL)

p-value calculated by Mann-Whitney *U* test

Table S4. Spearman correlation of elements between urine and fingernails

|  |  | Spearman correlation coefficient of elements between urine and nails | | | | | | | | | |
| --- | --- | --- | --- | --- | --- | --- | --- | --- | --- | --- | --- |
| Nail elements | **Li** | 0.24 | -0.04 | -0.05 | 0.03 | -0.12 | -0.12 | -0.08 | 0.03 | -0.07 | -0.04 |
|  | **Mg** | -0.06 | -0.07 | -0.16 | 0.02 | -0.00 | -0.10 | -0.07 | -0.04 | 0.05 | 0.00 |
|  | **Ni** | 0.00 | -0.15 | -0.09 | -0.01 | 0.01 | -0.05 | -0.08 | -0.08 | 0.09 | -0.07 |
|  | **Cu** | 0.08 | 0.02 | 0.04 | 0.08 | -0.03 | 0.15 | 0.11 | 0.07 | 0.25***** | -0.09 |
|  | **Zn** | -0.05 | -0.25* | -0.04 | -0.18 | -0.16 | -0.12 | -0.09 | -0.17 | -0.19 | -0.14 |
|  | **As** | 0.19 | 0.13 | 0.24 | 0.04 | 0.01 | -0.06 | 0.03 | 0.33* | 0.63* | 0.08 |
|  | **Se** | 0.14 | 0.06 | -0.18 | 0.17 | 0.11 | 0.08 | 0.10 | 0.01 | -0.13 | 0.11 |
|  | **Sr** | -0.13 | 0.01 | -0.08 | -0.01 | -0.07 | -0.15 | -0.18 | 0.06 | 0.21 | 0.05 |
|  | **Mo** | 0.02 | -0.23 | -0.01 | 0.03 | -0.08 | -0.09 | -0.05 | -0.16 | -0.02 | 0.05 |
|  | **Sn** | -0.08 | -0.14 | -0.04 | -0.08 | -0.14 | -0.13 | -0.15 | -0.12 | -0.09 | -0.08 |
|  |  | **Li** | **Mg** | **Ni** | **Cu** | **Zn** | **As** | **Se** | **Sr** | **Mo** | **Sn** |
|  |  | **Urinary element** | | | | | | | | | |

* p-value < 0.05

Table S5. Prediction performance of single element model trained by fingernail elemental contents

| Element | AUC | Sensitivity | Accuracy |
| --- | --- | --- | --- |
| Ni | 0.63 | 0.60 | 0.63 |
| Cu | 0.73 | 0.72 | 0.73 |
| Se | 0.61 | 0.64 | 0.61 |
| Sn | 0.57 | 0.48 | 0.57 |

AUC, area under the receiver operating characteristics curve.

Table S6. Prediction performance of multi-element model with basic characteristics trained by fingernail elemental contents

| No. of characteristic in group | Characteristic | AUC | Sensitivity | Accuracy |
| --- | --- | --- | --- | --- |
| 6 | A, B, PS, PAP, P, F | 0.66 | 0.01 | 0.51 |
| 5 | A, PS, PAP, P, F | 0.74 | 0.00 | 0.51 |
| 4 | PS, PAP, P, F | 0.78 | 0.00 | 0.51 |
| 3 | A, PS, F | 0.79 | 0.07 | 0.55 |
| 2 | A, B | 0.74 | 0.48 | 0.64 |
| 1 | A | 0.80 | 0.50 | 0.71 |

Ni, Cu and Se were included as predictors in all combinations.

A: age, B: BMI, PS: passive smoking, PAP: physical activity pattern, P: parity, F: family history of diabetes.

Table S7. Adjusted odd ratio (OR) of urinary elements for the risk of GDM

| **Element** | **OR (95%CI)** | **P-value** |
| --- | --- | --- |
| **Li** | 0.70 (0.37,1.29) | 0.261 |
| **Mg** | 0.89 (0.47, 1.69) | 0.729 |
| **Ni** | 0.88 (0.57, 1.34) | 0.556 |
| **Cu** | 0.90 (0.40, 2.01) | 0.796 |
| **Zn** | 1.55 (0.96, 2.67) | 0.089 |
| **As** | 0.82 (0.44, 1.49) | 0.514 |
| **Se** | 1.21 (0.63, 2.43) | 0.570 |
| **Sr** | 0.94 (0.45, 1.97) | 0.866 |
| **Mo** | 0.68 (0.34, 1.31) | 0.258 |
| **Sn** | 1.21 (0.76, 2.00) | 0.430 |

Odd ratio adjusted for education level, income, passive smoking, physical activity pattern, family history of diabetes, parity, age and pre-pregnancy BMI

Table S8. Adjusted odd ratio (OR) for the risk of GDM according to the tertiles of urinary elemental concentrations

| Element | Tertile | | OR (95% CI) | P-value | Element | Tertile | | OR (95% CI) | | P-value |
| --- | --- | --- | --- | --- | --- | --- | --- | --- | --- | --- |
| Li | Q1 | | ref | ref | As | Q1 | | ref | | ref |
|  | Q2 | | 0.36 (0.09, 1.35) | 0.140 |  | Q2 | | 0.54 (0.14, 2.06) | | 0.365 |
|  | Q3 | | 0.42 (0.10, 1.57) | 0.207 |  | Q3 | | 0.59 (0.16, 2.13) | | 0.416 |
| ***P for trend*** | | 0.243 | | | ***P for trend*** | | 0.394 | | | |
| Mg | Q1 | | ref | ref | Sr | Q1 | | ref | | ref |
|  | Q2 | | 0.32 (0.08, 1.22) | 0.104 |  | Q2 | | 0.28 (0.07, 1.09) | | 0.075 |
|  | Q3 | | 0.50 (0.12, 1.88) | 0.309 |  | Q3 | | 0.63 (0.16, 2.41) | | 0.501 |
| ***P for trend*** | | 0.279 | | | ***P for trend*** | | 0.446 | | | |
| Ni | Q1 | | ref | ref | Mo | Q1 | | ref | | ref |
|  | Q2 | | 0.25 (0.06, 0.96) | 0.051 |  | Q2 | | 0.61 (0.16, 2.25) | | 0.458 |
|  | Q3 | | 0.28 (0.07, 1.03) | 0.062 |  | Q3 | | 0.76 (0.19, 2.96) | | 0.687 |
| ***P for trend*** | | 0.084 | | | ***P for trend*** | | 0.647 | | | |
| Cu | Q1 | | ref | ref | Sn | Q1 | | ref | | ref |
|  | Q2 | | 0.47 (0.12, 1.79) | 0.274 |  | Q2 | | 1.81 (0.47, 7.43) | | 0.397 |
|  | Q3 | | 0.46 (0.11, 1.75) | 0.261 |  | Q3 | | 0.79 (0.20, 3.12) | | 0.737 |
| ***P for trend*** | | 0.233 | | | ***P for trend*** | | 0.888 | | | |
| Zn | Q1 | | ref | ref |  |  | |  | |  |
|  | Q2 | | 0.36 (0.09, 1.39) | 0.150 |  |  | |  | |  |
|  | Q3 | | 0.66 (0.17, 2.44) | 0.536 |  |  | |  | |  |
| ***P for trend*** | | 0.496 | | |  | |  | |  | |

Odd ratio adjusted for education level, income, passive smoking, physical activity pattern, family history of diabetes, parity, age and pre-pregnancy BMI

ref: reference group

Table S9. Prediction performance of single element model trained by urinary elemental contents

| Element | AUC | Sensitivity | Accuracy |
| --- | --- | --- | --- |
| Ni | 0.63 | 0.49 | 0.62 |
| Cu | 0.58 | 0.52 | 0.61 |
| Se | 0.58 | 0.50 | 0.58 |
| Sn | 0.59 | 0.65 | 0.64 |

AUC, area under the receiver operating characteristic curve.

Table S10. Prediction performance of multi-element model trained by urinary elemental contents

| No. of element in group | Element | AUC | Sensitivity | Accuracy |
| --- | --- | --- | --- | --- |
| 4 | Ni, Cu, Se, Sn | 0.62 | 0.43 | 0.60 |
| 3 | Cu, Se, Sn | 0.65 | 0.59 | 0.62 |
| 2 | Cu, Se | 0.65 | 0.53 | 0.67 |
| 1 | Ni | 0.63 | 0.49 | 0.62 |

AUC, area under the receiver operating characteristic curve.

Table S11. Prediction performance of multi-element model with basic characteristics trained by urinary elemental contents

| No. of characteristic in group | Characteristic | AUC | Sensitivity | Accuracy |
| --- | --- | --- | --- | --- |
| 6 | A, B, PS, PAP, P, F | 0.48 | 0.31 | 0.54 |
| 5 | B, PS, PAP, P, F | 0.59 | 0.43 | 0.55 |
| 4 | A, B, PS, P | 0.64 | 0.44 | 0.55 |
| 3 | B, PS, P | 0.72 | 0.72 | 0.75 |
| 2 | B, PS | 0.71 | 0.68 | 0.65 |
| 1 | PS | 0.66 | 0.62 | 0.62 |

Cu, Se, Sn were included as predictors in all combinations.

A: age, B: BMI, PS: passive smoking, PAP: physical activity pattern, P: parity, F: family history of diabetes.


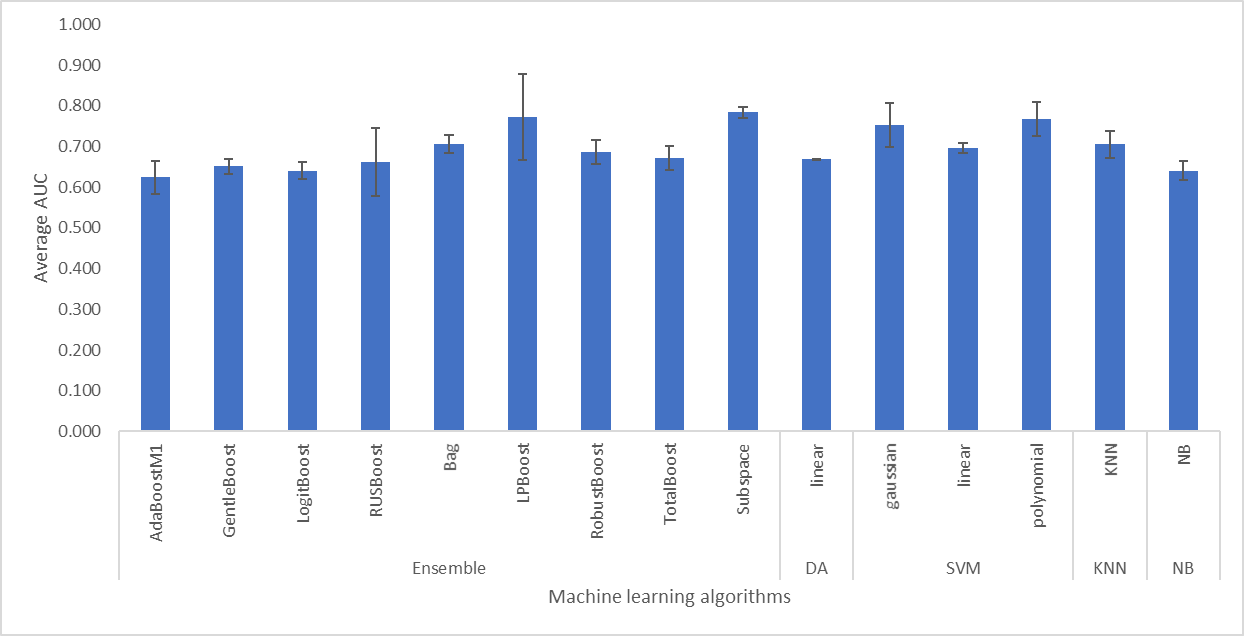


Figure S1. Comparison on the prediction performance among different trained models by fingernail elements


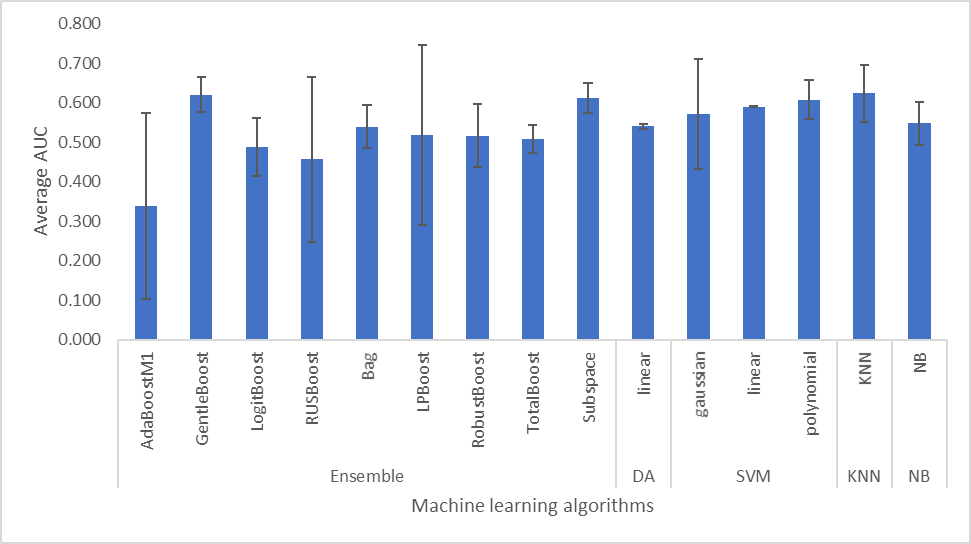


Figure S2. Comparison on the prediction performance among different trained models by urinary elements
